# Diabetes mellitus and hyperglycemia control on the risk of colorectal adenomatous polyps: a retrospective cohort study

**DOI:** 10.1186/s12875-018-0835-1

**Published:** 2018-08-29

**Authors:** Katarzyna Budzynska, Daniel Passerman, Denise White-Perkins, Della A. Rees, Jinping Xu, Lois Lamerato, Susan Schooley

**Affiliations:** 1 Department of Family Medicine, Henry Ford Hospital, Detroit, MI USA; 2 Department of Family Medicine and Public Health Sciences, Wayne State University, Detroit, MI USA; 3 Department of Family Medicine, Henry Ford Health System, 3370 E Jefferson, Detroit, MI 48207 USA

**Keywords:** Adenomatous polyp, Diabetes mellitus, Treatment, Colonoscopy

## Abstract

**Background:**

Colorectal cancer (CRC) develops from colorectal adenomatous polyps. This study is to determine if diabetes mellitus (DM), its treatment, and hemoglobin A1c (HbA1c) level are associated with increased risk of colorectal adenomatous polyps.

**Methods:**

This was a retrospective cohort study that included patients who had at least one colonoscopy and were continuously enrolled in a single managed care organization during a 10-year period (2002–2012). Of these patients (*N* = 11,933), 1800 were randomly selected for chart review to examine the details of colonoscopy and pathology findings and to confirm the diagnosis of DM. Multivariable logistic regression analyses were performed to assess the associations between DM, its treatment, HbA1c level and adenomatous polyps (our main outcome).

**Results:**

Among the total of 11,933 patients with a mean (standard deviation) age of 56 (± 8.8) years, 2306 (19.3%) had DM and 75 (0.6%) had CRC. Among the 1800 under chart review, 445 (24.7%) had DM, 11 (0.6%) had CRC and 537 (29.8%) had adenomatous polyps. In bivariate analysis, patients with DM had 1.45 odds of developing adenomatous polyps compared to those without DM. This effect was attenuated (odds ratio = 1.25, 95% CI: 0.96–1.62, *p* = 0.09) after adjusting for confounders such as age, gender, race/ethnicity, and body mass index. There was no significant association between type or duration of DM treatment or HbA1c level and adenomatous polyps.

**Conclusions:**

Our study confirmed the known increased risk of adenomatous polyps with advancing age, male gender, Hispanic race/ethnicity and higher body mass index. Although it suggested an association between DM and adenomatous polyps, a statistically significant association was not observed after controlling for other potential confounders. Further studies with a larger sample size are needed to further elucidate this relationship.

## Background

Colorectal cancer (CRC) develops from colorectal adenomatous polyps. It is estimated that there will be 134,490 new cases of CRC in 2016, 49,190 of those diagnosed will die [1, 2]. Screening colonoscopy prevents development of CRC by removal of precursor adenomatous polyps [3]. Since it takes between 7 and 10 years for the precancerous polyp to develop into a malignant lesion, routine screening colonoscopy has been shown to reduce the incidence of CRC and its subsequent morbidity and mortality [4]. However, despite advances in CRC screening and treatment modalities, CRC continues to be a leading cause of mortality in the United States. This highlights the need for more targeted interventions.

Diabetes mellitus (DM) has been found to be associated with an increased risk of CRC [2]. Several meta-analyses suggested that DM carries an average 30% increased risk of CRC [5–8]. It has been hypothesized that insulin resistance and the resulting hyperinsulinemia may promote carcinogenesis by directly stimulating colonic cell growth [9, 10]. In addition, insulin is thought to act indirectly by binding to and activating the insulin-like growth factor-1 receptors. Insulin-like growth factor-1 then enhances cell proliferation and inhibits apoptosis [9–12]. Observational studies have shown an increased CRC risk with hyperinsulinemia and elevated insulin-like growth factor-1 levels [13]. This is concerning as the number of Americans with DM has tripled over the last 3 decades [14]. The Centers for Disease Control and Prevention estimates that a total of 29.1 million Americans have DM and 29% of them are undiagnosed [14]. Additionally, it is even more concerning as African American ethnicity is identified as a risk factor for both Diabetes and CRC [15]. African Americans, among other minorities, have a higher prevalence and greater burden of diabetes, and lower screening rates for CRC [16].

Although there are strong data suggesting the association between DM and CRC, the current literature regarding the association between DM and adenomatous polyps, the precursor to CRC, is conflicting and has several limitations [17–20]. Some studies only evaluated a small sample with a short duration of exposure [21, 22] and other studies were conducted outside the United States [6]. In addition, there are only three studies that have evaluated the effect of glycemic control on the risk of adenomatous polyps with conflictual findings [17, 20, 21]. The main goal of this study was to better understand the association between DM and the prevalence of adenomatous polyps in a large managed care organization population. Additionally, the associations between the type of DM treatment (oral medicine vs. insulin), level of glycemic control (i.e., hemoglobin A1c [HbA1c] level), and the prevalence of adenomatous polyps were assessed. The study hypotheses were that: 1) patients with DM have increased prevalence of adenomatous polyps compared to those without DM; 2) higher HbA1c level is associated with higher risk of adenomatous polyps.

## Methods

### Study population

**Fig. 1:**
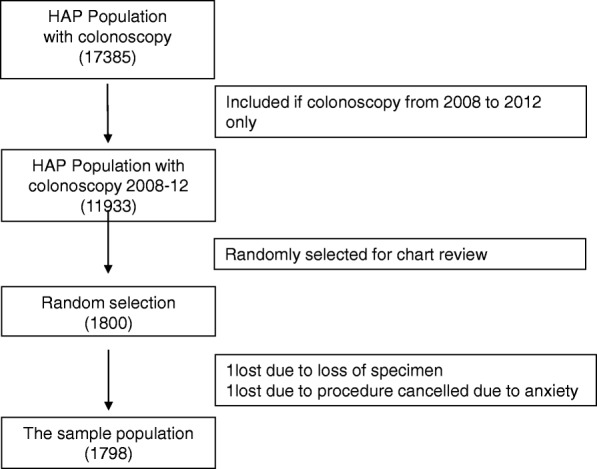
Inclusion and exclusion of study participants. Abbreviations: HAP, Health Alliance Plan

The initial population was identified by using an administrative database of a single managed care organization (i.e. Health Alliance Plan [HAP]) owned and operated by the Henry Ford Health System (HFHS). HFHS is a large metropolitan health system that spans 3 counties in southeast Michigan, including the city of Detroit. The study inclusion criteria were: 1) adult patients (≥ 18 years); 2) continuously enrolled in HAP for 10 years, who 3) had a colonoscopy during the second half of the 10-year period for either screening or diagnostic purposes. A total of 11,933 eligible patients were identified within 10 years (January 1, 2002 through December 31, 2012) who received care within the HFHS (Fig. 1). Of them, 1800 patients (the sample population) were randomly selected for medical record review (SPSS software) in 2015. The medical records were then reviewed to determine type of diabetes (type 1 vs type 2) and to identify presence, number, and types of polyp per pathology report (e.g., non-adenomatous polyp, adenomatous polyp, or CRC). The HFHS Institutional Review Board approved this study.

### Measurements of main exposure and outcome variables

The main outcome variable was presence of adenomatous polyps. All of the adenomatous polyps were identified by reviewing pathology reports of the colonoscopies. The main independent variable was having the diagnosis of DM within study period (January 1, 2002 to December 31, 2012). DM was categorized further to type 1 or type 2. In the initial total population (*N* = 11,933), a DM diagnosis was based on variables collected in the administrative data using factors employed by the Healthcare Effectiveness Data and Information Set (HEDIS) criteria [23], a long established metric for evaluating care of DM patients. The HEDIS criteria include the use of DM medications as well as DM codes, a methodology that reduces the prevalence of false positive diagnoses compared to the use of diagnostic codes alone [24].

For the sample population (*n* = 1800) that randomly selected for chart review, the diagnosis of DM was determined using information available from the medical record (e.g. fasting plasma glucose ≥126 mg/dl, plasma glucose ≥200 mg/dl at 2 h after a 75 g oral glucose load, HbA1c ≥ 6.5%, presence of medication used to treat DM, presence of insulin antibodies, or office notes indicating a diagnosis of DM).

### Covariate assessment

Data on additional covariates were collected from administrative databases of HFHS, including demographic information (age, gender, and race/ethnicity), body mass index (BMI) and HbA1c level. Age was categorized into quintiles (≤ 50, 51–55, 56–60, and >60 years, Table 1). BMI was calculated using height and weight measures and categorized based on World Health Organization criteria (normal BMI < 25, overweight 25 ≥ BMI < 30 kg/m^2^, or obese BMI ≥ 30 kg/m^2^) [25]. We did not separate underweight patients (BMI < 18.5 kg/m^2^) from normal weight patients due to its small percentage. HbA1c was used to represent the level of hyperglycemia control and it was categorized as 4 levels (normal HbA1c < 5.7, pre-DM 5.7–6.4, DM 6.5–7.9, and uncontrolled DM > 7.9) [23]. If more than one value of BMI and HgbA1c level were available in the chart, the median of each variable was used in the model. Type and length of medication exposure for oral antidiabetic medications and insulin were determined from filled prescriptions of drugs from HAP database, using the number of months of prescriptions filled prior to the date of colonoscopy, which was categorized as none, < 2 years, and ≥ 2 years.

**Table 1:** Characteristics of the population and chart review sample

Variables	Population (*N* = 11,933) N (%)	Chart review sample (*N* = 1798) N (%)	*p* value
Age, years, mean (SD)	56.06 (8.77)	56.20 (9.09)	0.468
≤ 50	2036 (17.1)	290 (16.1)	
51~ 55	4149 (34.8)	628 (34.9)	
56~ 60	3220 (27.0)	509 (28.3)	
> 60	2528 (21.2)	371 (20.6)	
Gender			
Female	6527(54.7)	964 (53.6)	0.317
Male	5406 (45.3)	834 (46.4)	
Ethnicity			
Caucasian	5794 (48.6)	874 (48.6)	0.544
African American	3572 (29.9)	528 (29.4)	
Hispanic	2148 (18.0)	342 (19.0)	
Asian	264 (2.2)	35 (1.9)	
Other/unknown	155 (1.3)	19 (1.1)	
Body mass index, kg/m^2^ mean (SD)	31.05 (6.86)	31.05 (7.05)	0.985
< 18.5	34 (0.3)	6 (0.3)	
18.5–24.9	1660 (13.9)	255 (14.2)	
25–29.9	3550 (29.7)	529 (29.4)	
30–39.9	4038 (33.8)	602 (33.5)	
> 40	1008 (8.4)	153 (8.5)	
Missing cases	1643 (13.8)	253 (14.1)	
Diabetes			
No	9627 (80.7)	1353 (75.3)	<.0001
Yes	2306 (19.3)	445 (24.7)	

*SD* standard deviation

*P* value obtained from chi-square test

### Statistical analyses

Sample characteristics were described using mean and standard deviations for continuous variables and frequencies (numbers and percentages) for categorical variables. Bivariate analysis was used to examine the effect of each of the covariates on prevalence of adenomatous polyps using chi-square test. Multivariable logistic regression models were used to examine the associations between the main exposure variable, DM, and the outcome variable, adenomatous polyps, adjusted for all other covariates. All analyses were performed using SPSS (IBM version of SPSS Statistics 2015).

## Results

A total of 11,933 patients met the study eligibility criteria (Fig. 1). Of them, a sample of 1800 patients was randomly selected for medical record review and this is called “the sample population”. Two participants were excluded from the sample population analysis: one due to loss of the pathology specimen and one due to the colonoscopy not performed because of patient anxiety of the procedure and poor prep. The demographic characteristics of the sample population (*n* = 1798) were statistically similar to the total HAP population except that the prevalence of DM diagnosis was greater in the sample population compared to total population (*n* = 11,933) (Table 1). This is because more patients with DM (25%) were identified by medical record review compared to those identified by HEDIS criteria (19%) in the administrative database of HAP population (*p* < 0.0001) (Table 1). When we used HEDIS criteria to identify patients with DM in the sample population, we found the prevalence of DM was similar as in the HAP population (*P* = 0.27, data not shown), which confirms that our randomization was successful. The mean age of the population (*n* = 1798) was 56.2 (± 9.1) years with females comprising 54% of the sample. Eighty-one percent were between ages 50 and 64 years. Forty-nine percent were Caucasian, followed by 30% African-American, 19% Hispanic, 2% Asian and 1% other/unknown. Seventy-one percent of the population was overweight or obese, with a mean BMI of 31.1 kg/m2 (± 7.1) (Table 1).

In the bivariate analysis (Table 2), we found significant associations between the presence of adenomatous polyps and older age (*p* < 0.0001), male gender (*p* < 0.001), higher BMI (*p* = 0.004), and a diagnosis of DM (*p* = 0.001). We found no significant association between presence of adenomatous polyps and race/ethnicity or HbA1c level. Additionally, among patients with DM, only age and gender were found to be associated with adenomatous polyps. Being on insulin or just taking oral DM medications or the length of DM treatment were not associated with adenomatous polyps. There were only seven people with type 1 DM in the subsample, thus no further analysis was done in this group.

**Table 2:** Characteristics among chart review sample and diabetes mellitus sample by adenomatous polyps

Variables	Adenomatous polyp among chart review sample			
	Total N (%)	No (*N* = 1261) N (%)	Yes (*N* = 537) N (%)	*P* value
Age, years, mean (SD)	56.20 (9.09)	55.39 (9.12)	58.09 (8.75)	< 0.0001
≤ 50	290 (16.1)	233 (80.3)	57 (19.7)	
51~ 55	628 (34.9)	441 (70.2)	187 (29.8)	
56~ 60	509 (28.3)	347 (68.2)	162 (31.8)	
> 60	371 (20.6)	233 (62.8)	138 (37.2)	
Gender				
Female	964 (53.6)	712 (73.9)	252 (26.1)	< 0.0001
Male	834 (46.4)	549 (65.8)	285 (34.2)	
Ethnicity				
Caucasian	874 (48.6)	624 (71.4)	250 (28.6)	0.131
African American	528 (29.4)	380 (72.0)	148 (28.0)	
Hispanic	342 (19.0)	220 (64.3)	122 (35.7)	
Asian	35 (1.9)	24 (68.6)	11 (31.4)	
Other/unknown	19 (1.1)	13 (68.4)	6 (31.6)	
Body mass index, kg/m^2^, mean (SD)	31.05 (7.06)	30.70 (7.04)	31.83 (7.03)	0.004
< 18.5	6 (0.3)	6 (100.0)	0 (0.0)	0.013
18.5–24.9	255 (14.2)	196 (76.9)	59 (23.1)	
25–29.9	529 (29.4)	369 (69.8)	160 (30.2)	
30–39.9	855 (47.6)	405 (67.3)	197 (32.7)	
> 40	153 (8.5)	98 (64.1)	55 (35.9)	
Diabetes				
No	1353 (75.3)	976 (72.1)	377 (27.9)	0.001
Yes	445 (24.7)	285 (64.0)	160 (36.0)	
	Adenomatous polyp among patient with Diabetes Mellitus (*N* = 445)			
Age, years, mean (SD)	58.76 (9.72)	57.54 (9.34)	60.91 (10.03)	0.001
≤ 50	45 (10.1)	35 (77.8)	10 (22.2)	
51~ 55	133 (29.9)	89 (66.9)	44 (33.1)	
56~ 60	128 (28.8)	82 (64.1)	46 (35.9)	
> 60	139 (31.2)	78 (56.1)	61 (43.9)	
Gender				
Female	225(50.6)	155(68.9)	70(31.1)	0.024
Male	220(49.4)	129(58.6)	91(41.4)	
Ethnicity				
Caucasian	169 (38.0)	107 (63.3)	62 (36.7)	0.622
African American	177 (39.8)	119 (67.2)	58 (32.8)	
Hispanic	80 (18.0)	46 (57.5)	34 (42.5)	
Asian	12 (2.7)	7 (58.3)	5 (41.7)	
Other/unknown	7 (1.6)	5 (71.4)	2 (28.6)	
Body mass index, kg/m^2^, mean (SD)	34.24 (7.54)	34.28 (7.47)	34.18 (7.67)	0.903
< 18.5	2 (0.4)	1 (50.0)	1 (50.0)	
18.5–24.9	26 (5.8)	15 (57.7)	11 (42.3)	
25–29.9	90 (20.2)	54 (60.0)	36 (40.0)	
30–39.9	205 (46.1)	135 (65.9)	70 (34.1)	
> 40	71 (16.0)	43 (60.6)	28 (39.4)	
Hemoglobin A1c				
< 5.7	15 (3.4)	10 (66.7)	5 (33.3)	0.903
5.7–6.4	126 (28.3)	78 (61.9)	48 (38.1)	
6.5–7.9	214 (48.1)	140 (65.4)	74 (34.6)	
≥ 8.0	71 (16.0)	47 (66.2)	24 (33.8)	
Missing cases	19 (4.3)			
Oral medication exposure				
None	204 (45.8)	130 (63.7)	74 (36.3)	0.916
< 2 years	105 (23.6)	69 (65.7)	36 (34.3)	
≥ 2 years	136 (30.6)	86 (63.2)	50 (36.8)	
Insulin exposure				
None	371 (83.4)	237 (63.9)	134 (36.1)	0.963
< 2 years	24 (5.4)	16 (66.7)	8 (33.3)	
≥ 2 years	50 (11.20	32 (64.0)	18 (36.0)	

*SD* standard deviation

In multivariable logistic regression analysis, while the point estimate suggested an increase in odds of adenomatous polyps in patients with DM when compared to those without DM, this association was not statistically significant (*p* = 0.09) after adjusting for age, gender, race/ethnicity, and BMI (Table 3). There was no significant association between HbA1c level and adenomatous polyps when controlling for other factors, such as BMI. In secondary analysis of the subsample that only included patients with DM, neither the type of treatment (insulin vs. oral medications) nor length of treatment (none, < 2 years, or ≥ 2 years) was associated with adenomatous polyps (Table 4).

**Table 3:** Multivariable logistic regression predicting adenomatous polyps among chart review sample (*N* = 1798)

	Odds Ratio	95% CI		*P* value
		Lower	Upper	
Age (ref: ≤50 years)				
51~ 55	1.97	1.35	2.86	< 0.0001
56~ 60	2.00	1.36	2.94	< 0.0001
> 60	2.59	1.74	3.86	< 0.0001
Sex (ref: Female)	1.45	1.16	1.81	< 0.0001
Ethnicity (ref: Caucasian)				
African American	0.98	0.76	1.28	0.891
Hispanic	1.44	1.07	1.93	0.016
Asian	1.08	0.48	2.46	0.847
Other/unknown	1.08	0.40	2.94	0.879
Body mass index	1.02	1.00	1.04	0.022
Diabetes (ref: No)	1.25	0.97	1.62	0.091

*ref* reference group

**Table 4:** Multivariable logistic regression predicting colorectal adenomatous polyps among diabetes mellitus patients only (N = 445)

	Odds Ratio	95% CI		*P* value
		Lower	Upper	
Model 1				
Age (ref: ≤50)				
51~ 55	1.82	0.78	4.25	0.164
56~ 60	1.92	0.82	4.47	0.132
> 60	2.93	1.26	6.85	0.013
Sex (ref: Female)	1.46	0.95	2.24	0.084
Ethnicity (ref: Caucasian)				
African American	0.84	0.52	1.36	0.479
Hispanic	1.25	0.69	2.28	0.463
Asian	1.36	0.33	5.63	0.673
Other/unknown	0.78	0.14	4.37	0.776
Body mass index	1.01	0.98	1.04	0.437
Oral medication exposure (ref: None)				
< 2 years	0.81	0.47	1.39	0.440
≥ 2 years	0.91	0.56	1.49	0.716
Model 2				
Age (ref: ≤50)				
51~ 55	1.80	0.77	4.18	0.175
56~ 60	1.88	0.81	4.38	0.145
> 60	2.91	1.25	6.78	0.013
Sex (ref: female)	1.44	0.94	2.21	0.093
Ethnicity (ref: Caucasian)				
African American	0.84	0.52	1.36	0.480
Hispanic	1.24	0.68	2.26	0.477
Asian	1.27	0.31	5.23	0.738
Other/unknown	0.75	0.13	4.20	0.741
Body mass index	1.01	0.98	1.04	0.447
Insulin exposure (ref: None)				
< 2 years	1.01	0.40	2.54	0.978
≥ 2 years	1.03	0.53	1.99	0.938
Model 3				
Age (ref: ≤50)				
51~ 55	1.85	0.73	4.72	0.196
56~ 60	2.28	0.90	5.77	0.081
> 60	3.58	1.42	9.06	0.007
Sex (ref: female)	1.47	0.94	2.30	0.092
Ethnicity (ref: Caucasian)				
African American	0.82	0.49	1.35	0.432
Hispanic	1.24	0.67	2.30	0.497
Asian	1.32	0.32	5.48	0.701
Other/unknown	0.75	0.13	4.27	0.747
Body mass index	1.01	0.98	1.04	0.483
Hemoglobin A1c (ref: < 5.7)				
5.7–6.4	0.91	0.28	3.00	0.880
6.5–7.9	0.99	0.31	3.18	0.992
≥ 8.0	1.03	0.30	3.61	0.962

Model 1 is predicting polyps among diabetic patients on oral medication only; model 2 is predicting polyps among diabetic patients on insulin; and model 3 is predicting polyps among diabetic patients with different levels of Hemoglobin A1c level

## Discussion

In this retrospective cohort study, we found higher prevalence of colonoscopy-confirmed colorectal adenomatous polyps with older age, male gender, and higher BMI. Although having DM was significantly associated with higher prevalence of adenomatous polyps in the bivariate analysis, the association was attenuated in multivariable logistic regression after controlling for age, gender, BMI, and race/ethnicity. The odds ratio (1.25) was similar in value to that of published data (1.30), but it did not reach statistical significance (*P* = 0.09). In the subsample that included only patients with DM, we did not find any significant associations between HbA1c level, type or duration of DM treatment and prevalence of adenomatous polyps.

The current literature is conflicting regarding the link between DM and colorectal adenomatous polyps. For example, Dash et al. [19], in a nested case-control study (917 cases and 2751 controls) among the Black Women’s Health study, found no overall association between DM and risk of adenomatous polyps. In contrast, Suh et al. [6], in a retrospective study of 3505 patients in South Korea reported that patients with DM had a higher proportion of adenomatous polyps. Additionally, Eddi et al. [17], in a case-control study (261 cases and 522 matched controls) in the United States, found an increased risk between DM and colorectal adenomatous polyps. The reason for these contrasting results are not entirely clear but could be related to differences in the study design, population studied, and measurement and control of potential confounders such as BMI, dietary pattern and length of study follow-up. Further prospective cohort studies with a longer follow-up would be needed to clarify these issues.

Current literature is also conflicting regarding the link between glucose level and risk of colorectal adenomatous polyps. We did not find a significant association between HbA1c level and the risk of adenomatous polyps, which is consistent with that reported by multiple investigators [6, 17–19]. In contrast, Siddiqui et al. reported, in a retrospective study with 652 male patients, that diabetic patients with poor glycemic control had a significantly higher prevalence of right-sided adenomatous polyps [19]. This controversy may be a result of the inherent limitation of HbA1c level to reflect the duration or degree of hyperinsulinemia. Though it’s a measure of glycemic control in DM patients, HbA1c level cannot be directly translated to the length or extent of hyperinsulinemia. Hyperinsulinemia appears to be a carcinogenic as well as the insulin-like growth factors (IGF) [26, 27]. Further research is needed to investigate this relationship.

The findings of this study confirmed that older age, male gender, and higher BMI were associated with the adenomatous polyps. However, no significant association between type or duration of DM treatment and adenomatous polyp was found as some studies suggested [17, 19]. For example, metformin has been reported to decrease colon adenomatous polyps while insulin therapy may increase them [28–30]. In this study, we could not separate metformin treatment from the use of other oral medications. In addition, the possible protective effect of metformin or the increased risk of insulin on colon adenomatous polyps remains very controversial [17, 19, 21, 26, 31]. While African-American race has been reported as a risk factor for colorectal cancer, data is limited regarding its association with precancerous polyps [32–37]. Our study did not find any significant association between African-American race and adenomatous polyps. Further studies are needed in this area as well.

This study has a number of strengths. It was a large retrospective cohort study of patients continually enrolled in a closed managed care organization for 10 years, with a diverse mix of race/ethnicity. The entire cohort had at least one colonoscopy and the identification of colorectal cancer and adenomatous polyp in the subsample were confirmed by reviewing colonoscopy pathology reports. In addition, the diagnosis of DM was confirmed by chart review, as compared to some of the previous studies where DM was self-reported. This study population that had been continuously enrolled in a closed managed care organization facilitated the inclusion of medication type and duration of DM treatment. Finally, only patients with a colonoscopy done in the second half of the 10-year study period were included in the study minimized the baseline heterogeneity of adenomatous polyp risk in our study population.

### Limitations

This study has several limitations. First, as a retrospective cohort study, the study was limited to data reliably found in medical records. Information regarding the possible confounders such as smoking, physical activity, alcohol use, the length of DM and family history of CRC were not available. Second, CRC was not analyzed since there were too few CRC cases in this sample. Third, the study population included only patients who had undergone colonoscopy, which may not be representative of the general population. While our study population was diverse in its mix of race/ethnicity, it cannot be generalized to the entire U.S. population, as this region has overrepresentation of African American race/ethnicity (U.S. census 13% vs and 29% our sample) and underrepresentation of Caucasians (U.S. census 77%, vs. 48% our sample). Finally, the study time frame would exclude any CRC or adenomatous polyp that may have been identified before or after the study period.

## Conclusions

Our study findings provide important information and context for future studies focusing on the association between DM and adenomatous polyp and CRC. The relationships between demographics, BMI, DM and its treatment on the development of adenomatous polyps and subsequently CRC are very complex. Up to now, data are inconclusive regarding DM and adenomatous polyp. Determining the effect of these risk factors and their complex interactions with each other on the risk of adenomatous polyp would be invaluable to primary care physicians and public health policy makers. These findings would have potential implications for more targeted CRC screening in individuals with DM, thereby decreasing the incidence rate and mortality from CRC. Considering the high prevalence of type 2 DM in the United States, even a small increase in cancer risk could have considerable consequences at a population level.

## Abbreviations

BMI: Body mass index
CRC: Colorectal cancer
DM: Diabetes mellitus
HAP: Health Alliance Plan
HbA1c: Hemoglobin A1c
HEDIS: Healthcare Effectiveness Data and Information Set
HFHS: Henry Ford Health System
Ref: Reference group
SD: Standard deviation
